# Self-Standing Cutin Isolate Films

**DOI:** 10.3390/polym18131579

**Published:** 2026-06-25

**Authors:** Nevena Hromiš, Sandra Bučko, Zorica Stojanović, Senka Popović, Biljana Pajin, Milica Stožinić, Di Zhang, Nejra Omerović, Jaroslav Katona

**Affiliations:** 1 Faculty of Technology Novi Sad, University of Novi Sad, Bul. Cara Lazara 1, 21000 Novi Sad, Serbia; nevena.krkic@uns.ac.rs (N.H.); zorica.stojanovic@uns.ac.rs (Z.S.); madjarev@uns.ac.rs (S.P.); biljana.pajin@uns.ac.rs (B.P.); milica.stozinic@uns.ac.rs (M.S.); jaroslav.katona@uns.ac.rs (J.K.); 2 School of Food and Biological Engineering, Jiangsu University, Zhenjiang 212013, China; d.zhang@ujs.edu.cn; 3 BioSense Institute, University of Novi Sad, 21000 Novi Sad, Serbia; nejra@biosense.rs

**Keywords:** cutin isolate, cutin particles, pH dependent precipitation, biomaterials, cutin films, food packaging

## Abstract

Cutin, a natural polyester, has attracted attention as a precursor for bio-based materials mimicking plant cuticles, particularly in food packaging. Most studies focus on polycondensation of hydrolyzed cutin fractions or combining cutin hydrolysates with other components; however, cutin precipitation, conditions affecting it, and cutin isolate film properties, without addition of other filmogenic material, remain insufficiently understood. Owing to the pH-dependent solubility of cutin, which progressively decreases as pH is lowered from strongly alkaline to acidic conditions, this study investigates the influence of pH on cutin dispersion formation and characteristics, and evaluates the impact of these dispersion properties on the formation and performance of self-assembled cutin isolate films, with a view to developing films with improved water-barrier and moisture-resistance properties. The influence of three plasticizers, glycerol, propylene glycol, and polyethylene glycol 400, at two concentrations was also evaluated. Results demonstrated that pH is the primary factor influencing cutin isolate dispersion characteristics and film performance, with decreasing pH promoting cutin precipitation and particle aggregation, thereby inducing changes in film structure. The strongest effects were observed for swelling, solubility, and tensile strength, followed by water vapor permeability, elongation at break, and thickness. Plasticizer type mainly affected moisture content and significantly influenced permeability and thickness, while concentration of plasticizer primarily impacted permeability. Interactions between pH and plasticizer significantly influenced most properties. Films prepared from cutin dispersions at pH 6.5 and pH 5 with polyethylene glycol (10%) showed the best balance of mechanical and barrier properties. Additionally, films prepared from the cutin solutions at pH 12 with glycerol (20%) exhibited good mechanical performance and high solubility, suitable for specific applications.

## 1. Introduction

According to the United Nations Environment Program [1], the world is wasting food on an alarming scale, undermining food security and slowing progress toward a zero-waste, circular future. The UN “Food Waste Index” Report indicates that 1.05 billion tons of food were lost/wasted globally in 2022 [2]. Food loss and waste are major contributors to environmental degradation, accounting for up to 10% of global greenhouse gas emissions and up to 14% of methane emissions. Mitigating agrifood waste is recognized as one of the most economically viable and impactful climate interventions, in line with zero-waste strategies that prioritize prevention, resource efficiency, and systemic transformation. Circularity has been identified as a leading trend in the food sector for 2026. Agrifood waste valorization involves converting by-products, such as brewer’s spent grain, whey, oat hulls, fruit pomace, and potato protein, into value-added products. Side-stream valorization is becoming increasingly important in food technology, with waste streams being repurposed into ingredients, animal feed, fertilizers, or packaging materials. Furthermore, the elevated costs associated with producing bio-based alternatives to petroleum-derived plastics can be partially offset by utilizing agrifood waste streams [3].

Tomato (*Solanum lycopersicum*) is the second most produced and consumed vegetable crop in the world, after the potato. Most tomatoes are cultivated in Mediterranean countries and processed into products in which the seed and skin are removed, such as paste, sauce, juice, and ketchup [4]. This large processing industry generates substantial amounts of by-products, primarily skin and seeds, collectively referred to as tomato pomace. Tomato pomace accounts for 4–30% of the processed mass, corresponding to approximately 5–6.4 million tons of waste annually [5,6]. Traditionally, tomato pomace has been used as animal feed due to its relatively high protein content [7,8] or disposed of in landfills; however, it also represents a valuable biosource of sustainable chemicals and monomers. Recent research has focused on its valorization not only for animal nutrition and the extraction of antioxidants, such as lycopene, used in food, pharmaceuticals, and cosmetics industry [9,10], but also for the sustainable production of renewable materials, including cutin-based biopolymers [8,11,12,13,14,15]. Notably, tomato peel accounts for approximately 27% of tomato pomace and is particularly rich in cutin, which constitutes 40–80% of its dry weight, giving an estimated global production potential of 0.2–2.5 million tons of cutin annually [16].

Cutin is a polyfunctional biopolyester and one of the main structural components of the plant cuticle, the protective outer layer covering the aerial organs of plants [17]. It is predominantly composed of long-chain (C16 and C18) hydroxy fatty acids, among which dihydroxylated C16 fatty acids are the most abundant, often representing more than 60 wt.% of the total monomer composition [8,17]. These fatty acids are linked via ester bonds to form a high-molecular-weight, crosslinked polyester network. Cutin monomers are typically polyhydroxylated and contain both terminal and mid-chain hydroxyl groups. Additional functional groups may occur at the ω-position and along the mid-chain, including various oxygen-containing groups, such as epoxy, oxo, hydroxy, vicinal diol, and double bonds [17,18,19]. This structural diversity imparts cutin with a rigid three-dimensional matrix and significant polyfunctionality. In many plants, the cutin matrix is further associated with long-chain aliphatic compounds, mainly waxes, which further enhance the overall barrier properties of the cuticle. Together, these components protect the plant cuticle from mechanical and chemical damage and reduce the diffusion of oxygen and water. Due to its natural abundance, particularly in tomato peels, cutin can be readily recovered from agricultural by-products, making it a promising renewable resource for biopolymer development [4,8,11,19,20].

Cutin’s distinctive physicochemical characteristics make it a valuable precursor for the development of bio-based materials that mimic plant cuticles, particularly for sustainable food packaging applications [11,21]. However, isolating cutin from plant sources is challenging due to its insolubility and its incorporation into waxes and polysaccharides. Conventional extraction procedures typically involve sequential dewaxing, enzymatic removal of polysaccharides, and subsequent cutin isolation [22]. Although effective, these methods are resource-intensive and environmentally unfavorable. Chemical hydrolysis of dewaxed cuticles can release cutin monomers, but it often disrupts the polymer’s native structure and yields complex hydrolysates of monomers and oligomers. Recent progress has shifted toward the direct alkaline hydrolysis of cutin-rich agro-waste, such as tomato pomace, enabling efficient production of cutin monomers without prior dewaxing or enzymatic treatment [17,23]. This approach enhances scalability and environmental sustainability, thus supporting the industrial valorization of agricultural by-products.

Contemporary research on cutin-based materials primarily emphasizes the exploitation of hydrolysis-derived monomeric and oligomeric fractions, rather than the direct utilization of the native cutin polymer. Two predominant strategies have been established, namely polycondensation and self-assembly, with occasional combinatorial approaches. Polycondensation, the standard protocol for polyester synthesis [12,13,14,24,25], is based on the reaction of functional monomers at elevated temperature and under reduced pressure, in order to eliminate condensation by-products, such as water, thereby promoting polymerization [8]. In contrast, self-assembly harnesses non-covalent interactions—such as van der Waals forces—to direct the spontaneous organization of molecular entities into ordered supramolecular structures, including monolayers, multilayers, and more complex morphologies [26]. Because this process is highly sensitive to variables such as pH, temperature, and concentration, it offers the possibility of obtaining structurally defined assemblies under relatively mild conditions.

Several studies have reported the preparation of functional materials by combining cutin hydrolysates with other components through self-assembly [4,15,20,27,28]. However, to the best of our knowledge, the systematic analysis of the link between self-assembly synthesis conditions of cutin-based biomaterial and their resulting material properties has not been sufficiently investigated.

By varying the processing parameters used for cutin film formation, such as pH and concentration, it is possible to alter the charge and conformation of cutin monomers and oligomers obtained by alkaline hydrolysis, and consequently their interaction during self-assembly. In this study, the effects of pH and concentration on the interaction of cutin fractions and on the properties of the resulting synthesized cutin biomaterials were investigated. It is hypothesized that decreasing the pH of cutin dispersions from strongly alkaline to more acidic conditions reduces the magnitude of the negative zeta potential of cutin particles, thereby diminishing electrostatic repulsion and promoting particle aggregation and precipitation. The enhanced particle association is expected to facilitate cutin self-assembly during film formation, resulting in a denser and more hydrophobic film network with improved water-resistance properties.

## 2. Materials and Methods

### 2.1. Materials

Tomato (*Solanum lycopersicum*) peels were obtained from local Serbian farmers. Sodium hydroxide (NaOH) was obtained from Centrohem (Stara Pazova, Serbia). Hydrochloric acid (HCl, 36%) was purchased from Zorka (Šabac, Serbia) while hydrochloric acid (HCl, 30%), anhydrous sodium sulfate (Na_2_SO_4_), sulfuric acid (H_2_SO_4_, 96%) and potassium sulfate (K_2_SO_4_) were obtained from Merck (Darmstadt, Germany), and copper (II) sulfate pentahydrate (CuSO_4_·5H_2_O) and propylene glycol (p.a. 99.5%) were purchased from Centrohem (Stara Pazova, Serbia). Sodium methoxide (NaOCH_3_), polyethylene glycol 400 (PEG 400) and anhydrous methanol were obtained from Thermo Fisher Scientific (Waltham, MA, USA). Chloroform was purchased from BDH Chemicals (Leuven, France). Anhydrous pyridine and BSTFA containing 1% TMCS were obtained from Alfa Aesar, Thermo Fisher Scientific (Kandel, Germany). Nitrogen (N_2_) was supplied by Messer Tehnogas (Belgrade, Serbia). Dialysis tubing cellulose membrane (D9402, Typical molecular weight cut-off 14,000 Da) was obtained from Sigma Aldrich (St. Louis, MO, USA). Glycerol (99%) was purchased from NRK Inženjering (Belgrade, Serbia). Distilled water was used as a solvent. All reagents were of analytical grade.

### 2.2. Preparation of Cutin Isolate

Tomato peels were washed with tap water to remove residual seeds and juice and subsequently air-dried at room temperature. Cutin extraction was performed following the procedure described by Cigognini et al. [17]. Briefly, cutin was isolated from the dried tomato peels by alkaline extraction. The dried peels were first milled using a colloid mill and subsequently suspended in a 0.75 mol dm^−3^ NaOH solution at a tomato peels-to-solution mass-to-mass ratio of 1:12. The suspension was vigorously stirred using a mechanical stirrer at 90 °C for 2 h to facilitate cutin extraction.

The resulting dispersion was cooled to room temperature in a water bath and subsequently centrifuged at 10,000 rpm for 25 min. The supernatant was then filtered through quantitative filter paper to remove residual solids and obtain the cutin extract. Cutin was precipitated from the extract by the gradual addition of 6 mol dm^−3^ HCl until pH 5 was reached, resulting in the formation of a precipitated cutin dispersion.

The dispersion was stored at 4 °C overnight to ensure complete precipitation of cutin, after which it was centrifuged at 10,000 rpm for 25 min. The precipitated cutin was subsequently collected and redispersed in 300 mL of distilled water. The resulting dispersion was subjected to dialysis against distilled water using dialysis tubing cellulose membrane. The conductivity of the dispersion was continuously monitored throughout the dialysis process, decreasing from an initial value of 2.45 mS/cm to 200–300 μS/cm. As further decrease in conductivity became minimal beyond this point, the dialysis was terminated. Conductivity of the distilled water is ≈8 μS/cm.

The dialyzed cutin dispersion was cast into silicone molds and dried at room temperature until a constant mass of cutin isolate was achieved. The obtained dry cutin isolate was then ground using a colloid mill and stored in a sealed container protected from light at room temperature until further use.

### 2.3. Proximate Analysis

Moisture content was determined by the oven-drying method. Approximately 1.00 g of sample was accurately weighed into pre-dried and pre-weighed glass weighing bottles. During drying, the lids were kept slightly open to allow moisture release. The sample was dried at 105 °C to constant mass, cooled in a desiccator with the lids closed, and reweighed. Moisture content was calculated as the percentage mass loss and expressed on a wet basis (g/100 g sample).

Ash content was determined by dry ashing in a muffle furnace. About 1.00 g of the sample was placed into pre-weighed crucibles and gently carbonized over a low flame, followed by ashing in a muffle furnace at 550 °C for 4 h. After cooling in a desiccator, the crucibles were weighed. Ash content was calculated as the percentage of the residue remaining after complete incineration, and results were expressed on a dry weight basis (g/100 g DW).

Total lipid content was determined using Soxhlet extraction method. Approximately 5.00 g of dried sample was accurately weighed into a cellulose extraction thimble and extracted with n-hexane for 4 h using a Soxhlet apparatus. After extraction, the solvent was removed by evaporation, and the remaining lipid residue was dried to constant mass in an oven at 105 °C, cooled in a desiccator, and weighed. Lipid content was calculated as the percentage of the extracted lipids relative to the initial sample mass and expressed on a dry weight basis.

Total protein content of the cutin isolate was determined by the Kjeldahl method, which involves digestion, distillation, and titration to quantify the total nitrogen content. Approximately 0.10 g of sample was weighed into digestion tubes and digested with concentrated sulfuric acid in the presence of a catalyst mixture (e.g., CuSO_4_/K_2_SO_4_) until a clear digest was obtained. After cooling, the digest mixture was neutralized with 33% NaOH, and the released ammonia was distilled and collected in a 0.01 mol/dm^3^ hydrochloric acid solution. The excess acid was titrated with standardized 0.01 mol/dm^3^ NaOH. Total nitrogen content was calculated and converted to protein content using a conversion factor of 6.25.

The total dietary fiber content of the samples was determined in accordance with the standard AOAC Method No. 985.29 [29].

Residual fraction was calculated by difference as part of proximate composition analysis. Specifically, the residual fraction on a dry weight basis was estimated by subtracting the measured ash, fat, protein, and dietary fiber contents from 100%, according to

Carbohydrates Residual fraction (% DW) = 100 − (Ash + Fat + Protein + Dietary fiber)

The obtained value represents a calculated residual fraction that may include carbohydrates, cutin-associated material, and other non-quantified constituents. Values were calculated from the mean values of the measured components and reported as % DW.

All determinations were carried out in triplicate, and results are expressed as mean ± standard deviation. Moisture content is expressed on a wet basis, whereas all other components are expressed on a dry weight basis.

### 2.4. Depolimerization of Cutin

Depolymerization of the cutin isolate was performed by base-catalyzed methanolysis based on published procedures, with minor modifications [30,31,32]. Briefly, cutin isolate (0.025 g) was placed in an Erlenmeyer flask, and 3.0 mL of freshly prepared sodium methoxide (NaOCH_3_, 1 mol/dm^3^ in anhydrous methanol) was added. Moisture uptake was minimized by using anhydrous reagents and tightly sealed vessels. The mixture was incubated for 24 h at room temperature on a shaker (~500 rpm). After methanolysis, 2.0 mL of 2 mol/dm^3^ sulfuric acid in methanol was added to acidify the reaction mixture. The samples were centrifuged for 10 min at 3000 rpm, and the supernatant was collected. Subsequently, 10 mL of water was added, and the released monomers were extracted with chloroform (3 × 10 mL). Extractions were performed by vigorous shaking, and after phase separation, the lower organic phase was collected and combined in a clean tube. The combined chloroform extract containing the released monomers was dried over anhydrous Na_2_SO_4_, filtered, and the solvent was evaporated under a gentle stream of nitrogen. The yield of the depolymerized solvent-extractable fraction was determined gravimetrically, and expressed relative to the initial cutin isolate mass. When not analyzed immediately, the obtained cutin monomers were kept dissolved in chloroform and stored at −18 °C until further analysis.

### 2.5. GC–MS Analysis of Monomeric Compounds

Prior to GC–MS analysis, cutin monomer methyl esters were derivatized to their trimethylsilyl (TMS) derivatives, to improve volatility and chromatographic performance. Before derivatization, samples were dried to remove residual moisture by keeping them overnight in a desiccator. If the monomer extracts had been stored in the freezer, chloroform was first evaporated to dryness under nitrogen, and the residue was additionally dried overnight in a desiccator. Derivatization was performed by adding 100 μL of anhydrous pyridine and 100 μL of BSTFA containing 1% TMCS to the dried residue, followed by thorough mixing. The reaction mixture was incubated in an oven at 100 °C for 10 min and then allowed to cool to room temperature [32]. After derivatization, solvents evaporated to dryness under nitrogen, and the residue was reconstituted in 500 μL of hexane. The final solution was transferred into GC vials equipped with inserts and subjected to GC–MS analysis.

GC–MS analysis was performed on an Agilent Technologies (Palo Alto, Santa Clara, CA, USA) gas chromatograph GC 7890B (Palo Alto, Santa Clara, CA, USA) coupled to a 5977A mass-selective detector (Agilent Technologies, Palo Alto, Santa Clara, CA, USA) and autosampler 7693 (Agilent Technologies, Palo Alto, Santa Clara, CA, USA), using a HP-5ms Agilent Technologies capillary column (30 m × 0.25 mm, 0.25 µm). Helium was used as the carrier gas at a constant flow of 1.2 mL/min. Samples (1 μL) were injected at a split ratio of 10:1, with injector temperature set to 300 °C. The oven temperature program was set to enable separation of fatty acids, ω-hydroxy acids, and dicarboxylic acids: initial temperature 125 °C, ramped at 10 °C/min to 220 °C, then ramped at 3 °C/min to 290 °C and held for 10 min. The MS detector operated in electron ionization mode at 70 eV. The quadrupole temperature was 150 °C, the transfer line temperature 250 °C, and the solvent delay 1.8 min. Mass spectra were acquired in scan mode over an *m*/*z* range of 50–550. Compound identification was based on comparison of mass spectra with reference spectra in the Wiley 10th (Hoboken, NJ, USA) and NIST 2011 libraries and, when available, with authentic standards analyzed under the same conditions.

### 2.6. Cutin Isolate Dispersion Preparation

Cutin isolate dispersions were prepared by suspending the required amount of cutin isolate in 0.1 mol/dm^3^ NaOH solution to obtain dispersions with cutin isolate concentrations ranging from 1% to 10% (*w*/*w*). The dispersions were vigorously stirred using a magnetic stirrer for 24 h. Subsequently, the pH was adjusted to the desired value within the range of pH 3.5–8 by the gradual addition of 6 mol/dm^3^ HCl. After pH adjustment, stirring continued for an additional 24 h to allow the system to reach equilibrium.

### 2.7. Cutin Isolate Dispersion Characterization

The zeta potential (ζ) of cutin isolate dispersions (c1%, *w*/*w*) at different pH values was determined using a Zetasizer Nano ZS (Malvern Instruments, Worcestershire, UK). Measurements were performed using a folded capillary cell (DTS1060, Malvern Instruments, Worcestershire, UK). Cutin isolate dispersions with pH values ranging from 3.5 to 8 were diluted at a ratio of 1:50 using an appropriate dilution medium. The dilution medium consisted of 0.1 mol/dm^3^ NaOH solution adjusted to the corresponding pH by the addition of 6 mol/dm^3^ HCl. The pH of the diluted dispersions was verified prior to measurement and, if necessary, readjusted to the target value. All measurements were performed in triplicate.

The volume-weighted mean diameter (d_4,3_) of cutin isolate particles in the dispersions was determined using a Malvern Mastersizer Micro Particle Analyzer (Malvern Instruments Ltd., UK). Buffer solutions with pH values corresponding to those of the cutin isolate dispersions were used to obtain background measurements. The dispersions were dozed to the measurement cell to achieve an obscuration level between 10% and 20%. During the measurements, the pump rotation speed was maintained at 1500 rpm.

### 2.8. Cutin Isolate Film Preparation

Cutin isolate films were prepared from cutin isolate dispersions at pH 5 and pH 6.5, as well as from a cutin isolate solution at pH 12, all containing 10% (*w*/*w*) of cutin isolate. Films prepared from 1% and 5% cutin dispersions exhibited inadequate cohesion and mechanical integrity and could not be reliably isolated for characterization. Therefore, only films from 10% dispersions were further analyzed as the only experimentally viable formulation.

The cutin isolate solution was prepared by suspending cutin isolate in 0.35 mol/dm^3^ NaOH to obtain a final concentration of 10% (*w*/*w*), followed by vigorous stirring at a magnetic stirrer for 24 h to ensure complete dissolution.

After preparation of the cutin isolate dispersions and the cutin isolate solution, one of the three different plasticizers, namely glycerol (G), propylene glycol (PG), or polyethylene glycol 400 (PEG), was incorporated into the cutin isolate dispersion or solution. The plasticizers were added at concentrations of 10% (*w*/*w*) and 20% (*w*/*w*), calculated relatively to the mass of cutin isolate present in the dispersion or solution.

Following plasticizer addition, the cutin isolate systems were stirred for an additional hour to ensure uniform distribution of the plasticizer. The resulting film-forming systems were then cast into Teflon-coated molds, maintaining a constant casting mass of 0.14 g cm^−2^. The molds were left at room temperature for 48–72 h to allow solvent evaporation and cutin isolate film formation. Afterwards, films were stored in a refrigerator till analysis. Before analysis, the films were conditioned for 48 h at 23 ± 2 °C and 50 ± 5% relative humidity.

At pH 12, films without plasticizer and those containing 10% plasticizer were excessively brittle and could not be successfully peeled from the casting surface.

### 2.9. Cutin Isolate Film Characterization

Structural properties of the films were analyzed using Nicolet IS10 FTIR spectrophotometer (Thermo Fisher Scientific, Waltham, MA, USA) employing attenuation total reflection (ATR) technique. Spectra were recorded in the range 4000–400 cm^−1^ at a resolution of 4 cm^−1^ taking 16 scans per record. Before each sample measurement, background spectra were recorded. Omnic 8.1. (Thermo Fisher Scientific, Waltham, MA, USA) software was used to process collected FTIR spectra.

Film thickness was measured using a micrometer Digico 1 (Tesa, Renens, Switzerland) with a sensitivity of 0.001 mm according to ASTM D6988-21 [33]. Thickness was determined at 8 different points on each film.

For the determination of water content (WC) and film solubility in water, film samples were cut in square probes (20 mm × 20 mm) and weighed (w1). Film probes were then dried at 105 ± 2 °C to constant mass and reweighted (w2) [34]. WC was determined as follows:

(1) $$WC(\%)=\frac{w1-w2}{w1}*100$$

For the determination of film solubility in water, after measuring WC of films, films were immersed in 50 mL of distilled water and gently shaken for 24 h. Afterwards, films were removed from the water and dried at 105 ± 2 °C to constant weight (w3). Solubility of films in water was calculated as follows:

(2) $$Solubility\left(\%\right)=\frac{w2-w3}{w2}*100$$

The swelling degree of the films in water was determined using probes of films (10 mm × 20 mm). Probes were weighted (w1) and then immersed in deionised water at 23 °C for 2 min [35]. Films were then removed from the water, and excess water was absorbed gently using paper towels. The film probes were then reweighed (w2). The swelling degree was then calculated:

(3) $$Swelling\;degree\left(\%\right)=\frac{w2-w1}{w1}*100$$

The obtained values represent the initial water uptake of the films rather than equilibrium swelling.

Water vapor barrier properties of the films were determined gravimetrically by the dish method according to the ISO 2528:1995 [36] and ASTM 96 [37]. The test conditions were 23 ± 2 °C and 57 ± 1% relative humidity. A saturated sodium bromide solution was placed inside the cup, while dry silica gel was used in the outer atmosphere (0% RH). Average permeability (g/(m•s•Pa)) was calculated according to Equation (4):

(4) $$WVP=\frac{G•d}{t•3600•A•\Delta p}$$

G/t = slope of the straight line of the mass loss versus time, g/h; A—test area (cup mouth area) in m^2^, which in this case was 2.27·10^−4^ m^2^; d is the average thickness of the sample in m; and Δp = vapor pressure difference in Pa. The film thickness used for WVP calculations corresponded to the average value obtained from multiple measurements at different positions on each film sample. To prevent edge leakage, the film specimen was positioned between two silicone O-rings and tightly compressed by the screw-cap assembly of the permeability cup, ensuring a hermetic seal so that water vapor transmission occurred exclusively through the exposed film area.

Mechanical properties of the films, i.e., tensile strength (TS) and elongation at break (EB), were tested in accordance with the ASTM D882-18:2018 standard method on the Instron 4301 testing machine (Instron Engineering, Canton, MA, USA). Film pH 6.5/G20 and pH 12/PEG 20 could not be tested for mechanical properties due to the stickiness of the pH 6.5/G20 film and brittleness of the film pH 12/PEG20.

Summary of the prepared films, including explanations for samples excluded from further analysis owing to insufficient mechanical properties, is provided in Table 1.

For each sample and each analysis, measurements were performed on at least three independently prepared films.

The surface and cross-sectional morphologies of the films were examined using a Thermo Scientific ApreoTM 2C scanning electron microscope (Thermo Fisher Scientific, USA). Samples were mounted to aluminum stages using double-sided carbon adhesive tape. Images were acquired at a low accelerating voltage (1–2 kV).

### 2.10. Statistical Analysis

Statistical analysis of film properties was performed using Jamovi software (Version 2.7.30; The jamovi Project, Sydney, Australia). Due to the fact that some films could not be successfully obtained in required quality, some properties could not be measured for all samples (Table 2) and a three-way analysis of variance (ANOVA) could not be applied. Instead, two-way analysis of variance (ANOVA) was applied to evaluate the combined effects of pH and plasticizer type, as well as the effects of plasticizer type and concentration on the physicochemical and mechanical properties of the films. When significant effects were detected, Tukey’s post hoc test was used for pairwise comparisons between factor levels. Differences were considered statistically significant at *p* < 0.05. Interaction plots and estimated marginal means were generated to illustrate the effects of the studied factors on film properties.

## 3. Results and Discussion

### 3.1. Chemical Characterization of Cutin Isolate

The proximate composition of the tomato cutin isolate is presented in Table 3. The sample exhibited a low moisture content (2.54 ± 0.01%), which is desirable for storage stability. The ash content was 0.18 ± 0.05%, indicating a minor contribution of inorganic matter. The fat content was 12.12 ± 0.42%, suggesting the presence of residual extractable lipophilic compounds, such as cuticular waxes and other extractable lipids, which may remain in cutin-rich isolates unless exhaustive dewaxing is performed [22].

The protein content, determined by Kjeldahl method using a conversion factor of 6.25, was 7.53 ± 0.55%. The residual fraction, calculated by difference, constituted the majority of the isolate (63.02 ± 1.63%). This fraction most likely includes carbohydrates and polysaccharide-rich cell-wall residues together with cutin-associated material and other non-quantified constituents [38].

The relative standard deviations for most measured components were below 10%, indicating acceptable reproducibility of the isolate preparation and satisfactory sample homogeneity.

Beyond the basic compositional profile, the yield of the depolymerized solvent-extractable fraction of the tomato cutin isolate was additionally evaluated. The gravimetric yield obtained after methanolysis and chloroform extraction was 81.41 ± 5.69%, expressed relative to the initial cutin isolate mass. This fraction may include not only cutin-derived monomers and oligomers, but also co-extracted lipophilic constituents (e.g., cuticular waxes) and other extractables. This high yield indicates that a large portion of the isolate was converted into solvent-extractable depolymeriyation products under the applied conditions. Subsequently, GC–MS analysis was performed to identify and profile the cutin-derived monomers present in the depolymerized fraction.

### 3.2. GC–MS Profile of Cutin Monomers in Tomato Cutin Isolate

Results of GC/MS analysis of cutin-related monomers released by methanolysis are shown in Table 4. The depolymerized fraction was dominated by hydroxy fatty acids, which represent typical building blocks of cutin polyesters. In particular, 10,16-dihydroxyhexadecanoic acid was the predominant component, representing 70.02 ± 4.25% of the total chromatographic signal, consistent with previous studies reporting this compound as the main monomer in tomato cutin [38,39]. Together with 9,10,18-trihydroxy octadecanoic acid (1.13 ± 0.01%), the di- and tri-hydroxy fatty acids comprised 71.15% of the profile, indicating that the isolate was strongly enriched in cutin-related monomers. A smaller contribution of monohydroxy fatty acids was observed, with 16-hydroxy hexadecanoic acid comprising 2.60 ± 0.35%.

In addition to cutin-derived hydroxy acids, several fatty acid methyl esters and TMS derivatives (Σ 6.60%) were detected, which may reflect the co-extraction of residual lipophilic components (e.g., wax- or lipid-derived compounds) remaining in the cutin-enriched isolate. As plant cuticles contain cutin along with waxes and polysaccharides [38,39], non-cutin residues may persist in cutin-enriched isolates depending on purification extent. Two peaks were assigned to *p*-coumaric acid (total 1.63%), which may reflect different derivatized forms possibly with some co-elution effects under the applied GC conditions. A fraction of the chromatographic signal remained non-identified (9.94 ± 2.69%), likely due to low abundance and limited spectral match quality.

### 3.3. Cutin Isolate Dispersion Characterization

**Figure 1:**
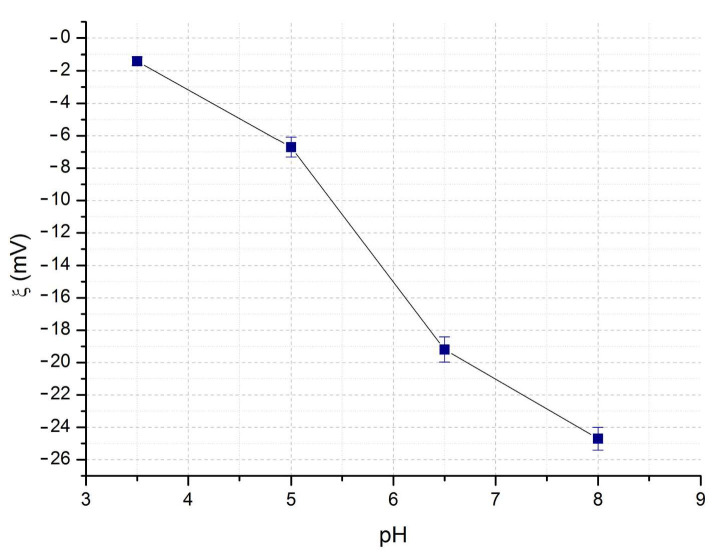
Influence of pH (3.5–8) on zeta potential (ζ) of cutin isolate dispersions.

Cutin isolate dispersions were formed by a controlled precipitation process induced by reducing the pH of the cutin solution from highly alkaline conditions (pH ≈ 13) to a predefined range of pH 3.5–8. The effect of pH on their zeta potential was investigated, and the results are illustrated in Figure 1.

Cutin isolate particles exhibit a negative zeta potential, the magnitude of which increased with increasing pH in the range of 3.5 to 8. Namely, at pH 3.5 the ξ was barely negative at −1.35 mV, while it reaches −24.1 mV at pH 8. This behavior can be attributed to the progressive ionization of carboxyl groups present in C16–C18 cutin-derived fatty acids that constitute the predominant structural components of cutin [4,19].

**Figure 2:**
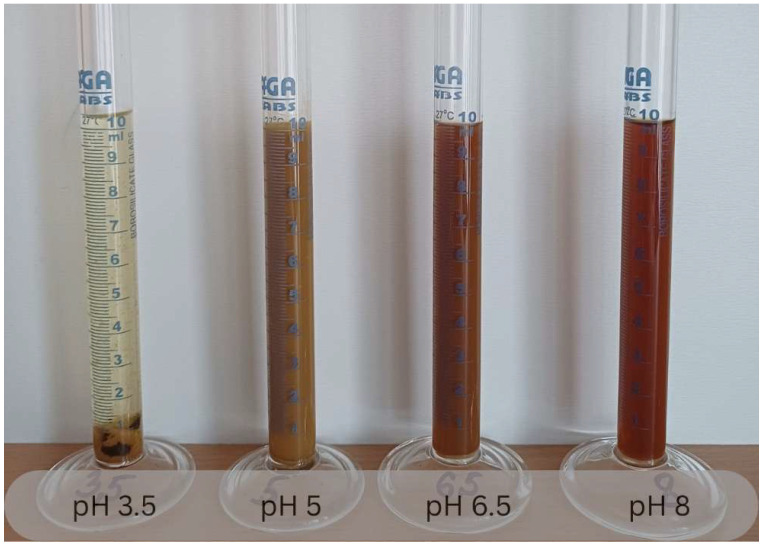
Influence of pH (3.5–8) on the appearance of cutin isolate dispersions, c = 1% (*w*/*w*).

The influence of the pH on the visual appearance of the cutin isolate dispersions is illustrated in Figure 2.

As shown in Figure 2, lowering the pH from alkaline to acidic conditions resulted in pronounced changes in the system’s appearance, progressing from a dark brown, transparent solution at higher pH values (pH 8) to a pale yellow, turbid dispersion at intermediate pH (e.g., pH 6.5), and ultimately to complete precipitation of cutin isolate particles accompanied by a clear supernatant at a lower pH. This transformation reflects the progressive precipitation of cutin and can be related to the corresponding decrease in the zeta potential of the particles (Figure 1). Namely, the reduction in electrostatic repulsion allows particles to approach one another more closely, thereby promoting particle aggregation and compromising dispersion stability. As electrostatic stabilization progressively diminishes, aggregation becomes increasingly favorable, ultimately leading to sedimentation and precipitation at pH values where electrostatic stabilization is minimal, i.e., ≈pH 3.

The pH-dependent flocculation and precipitation of cutin can be interpreted from the standpoint of cutin self-assembly, where particle aggregation may be advantageous for film formation. As cutin agregation is primarily governed by non-covalent interactions, particularly van der Waals and hydrophobic interactions, the enhanced particle association induced by decreasing pH may promote the formation of more compact and hydrophobic film networks. Consequently, controlled cutin aggregation may contribute to the development of films with improved water-resistance properties [20,40].

**Figure 3:**
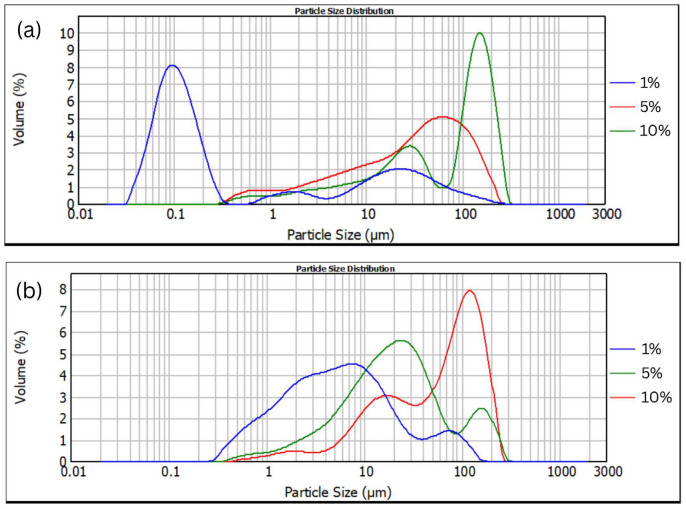
The influence of the cutin isolate concentration (1–10% (*w*/*w*)) on the volume weighted particle size distribution at (**a**) pH 5 and (**b**) pH 6.5.

For film-forming applications, only dispersions at pH 5 and pH 6.5 were considered suitable. Accordingly, particle size, as well as the effect of cutin dispersion concentration, were evaluated at these two pH values. The influence of cutin isolate dispersion concentration (1–10% *w*/*w*) on the volume-weighted particle size distribution at pH 5 and pH 6.5 is presented in Figure 3.

At both pH 5 and pH 6.5, increase in the cutin isolate concentration results in a shift in the volume-weighted diameter distribution toward larger particle sizes, with more pronounced shifts observed at higher concentrations. This behavior can be attributed to the increased viscosity of the cutin dispersions at elevated concentrations, which favors the formation of larger particles during the precipitation process [41].

The effect of cutin isolate concentration (1–10% *w*/*w*) on the mean volume-weighted particle diameter at pH 5 and pH 6.5 is presented in Table 5.

As shown in Table 5, the d_4,3_ values confirmed that particle diameter increased with increasing cutin isolate concentration. Furthermore, the difference in particle size between pH 5 and pH 6.5 becomes more pronounced at higher concentrations. This effect can be attributed to the increased electrostatic stability of cutin particles at elevated pH values due to higher zeta potential (Figure 1). Specifically, at pH ≥ 6.5, in comparison to the particle diameter at pH 5, it seems that the increased particle zeta potential enhances electrostatic repulsion, thereby inhibiting aggregation and limiting the formation of larger particles.

### 3.4. Cutin Isolate Film Characterization

Cutin isolate films were prepared by casting from a 10% (*w*/*w*) cutin isolate dispersion at pH 5 and pH 6.5, as well as from a cutin isolate solution at pH 12. Each casting medium was formulated both without plasticizer and with the addition of plasticizers at concentrations of 10% or 20% (based on the dry mass of cutin isolate). Three different plasticizers were employed: glycerol (G), propylene glycol (PG), and polyethylene glycol 400 (PEG).

**Figure 4:**
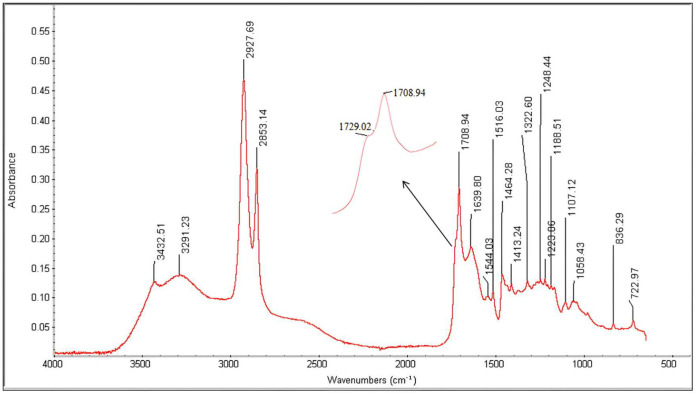
ATR-FTIR spectra of cutin izolate from tomato peels.

The ATR-FTIR spectra of the cutin isolate obtained from tomato peels is presented in Figure 4 and revealed multiple absorption bands.

A broad absorption band with two maxima at 3432 cm^−1^ and 3291 cm^−1^ corresponds to O–H stretching vibrations, with possible overlap from N–H stretching vibrations of protein residues. This band most probably originates from hydroxyl groups of cutin hydroxy-fatty acids, adsorbed water, some fiber, and residual proteins.

Two strong bands at 2927.69 cm^−1^ and 2853.14 cm^−1^ can be assigned to the asymmetric and symmetric stretching vibrations of CH2 groups, respectively. These peaks are followed by bands at 1464.28 cm^−1^, 1322.60 cm^−1^, and 722.97 cm^−1^, related to CH_2_ bending vibrations. Together, these bands indicate the presence of hydrophobic aliphatic constituents, mainly cutin and residual waxes. In the carbonyl region, a main band appears at 1708.94 cm^−1^, which is slightly lower than the typical position of the ester carbonyl group in cutin (around 1730 cm^−1^). This shift may be associated with the structural modifications induced by the alkaline treatment applied during isolation. The observed FTIR changes are in consistency with an increased contribution of carboxyl/carboxylate groups, which may result from partial hydrolysis of ester bonds in the cutin polyester network followed by protonation during acid precipitation with HCl. The presence of free COOH groups and hydrogen-bonded carbonyls could therefore contribute to a shift in the maximum of the carbonyl band towards lower wavenumbers. In addition, a shoulder at about 1729 cm^−1^ was observed, corresponding to ester carbonyl stretching vibrations characteristic of the cutin polyester structure. The coexistence of these two signals is consistent with the presence of both esterified and free carboxyl functional groups and may indicate partial hydrolysis of the cutin network. Peaks at 1248.44, 1229.06, and 1188.51 cm^−1^, as well as 1107.12 and 1058.43 cm^−1^, are also related to the cutin spectra, but could also arise from present polysaccharides. Peaks between 1650 and 1500 cm^−1^, as well as the peak at 836.29 cm^−1^, are attributed to phenolic compounds. However, the bands at 1639.80 cm^−1^ and 1544.03 cm^−1^ can also be related to protein residues and represent amide I and amide II bands, respectively [42].

**Figure 5:**
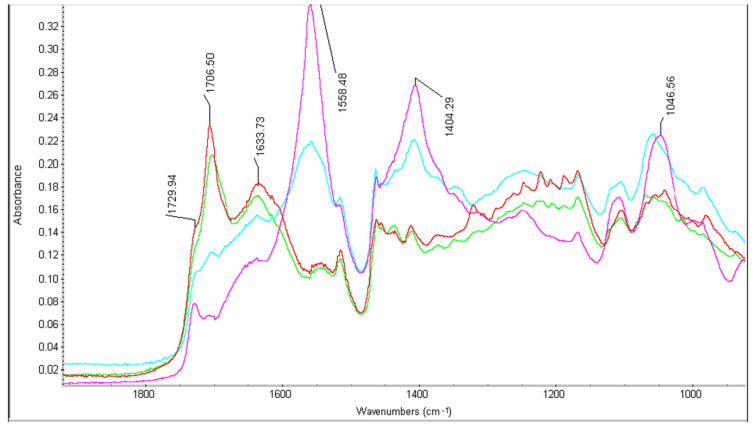
ATR FTIR spectra of cutin rich material isolated from tomato peels (red spectrum), film obtained at pH 5 (green spectrum), film obtained at pH 6.5 (blue spectrum) and film obtained at pH 12 (violet spectrum).

Looking at the fingerprint region for cutin (Figure 5), it can be noted that the spectrum of the cutin-rich isolate is similar to that of the film obtained at pH 5. The shoulder at 1729.94 cm^−1^ and the peak at 1706.50 cm^−1^ indicate that both the isolate and the pH 5 film consist of a partially hydrolysed cutin network containing both esterified and free carboxyl functional groups. The ATR-FTIR spectra reveal a pH-dependent transformation of the cutin-based matrix. With increasing pH, there is a progressive decrease in the carbonyl bands at 1729–1706 cm^−1^, accompanied by a strong increase in the bands at 1558 cm^−1^ (asymmetric stretching of COO^−^) and 1404 cm^−1^ (symmetric stretching of COO^−^) [43]. These changes are consistent with the conversion of protonated carboxylic groups into carboxylate species (COO^−^). The film prepared from cutin solution at pH 12 exhibited the most pronounced carboxylate features, indicating extensive alkaline modification of the matrix.

**Figure 6:**
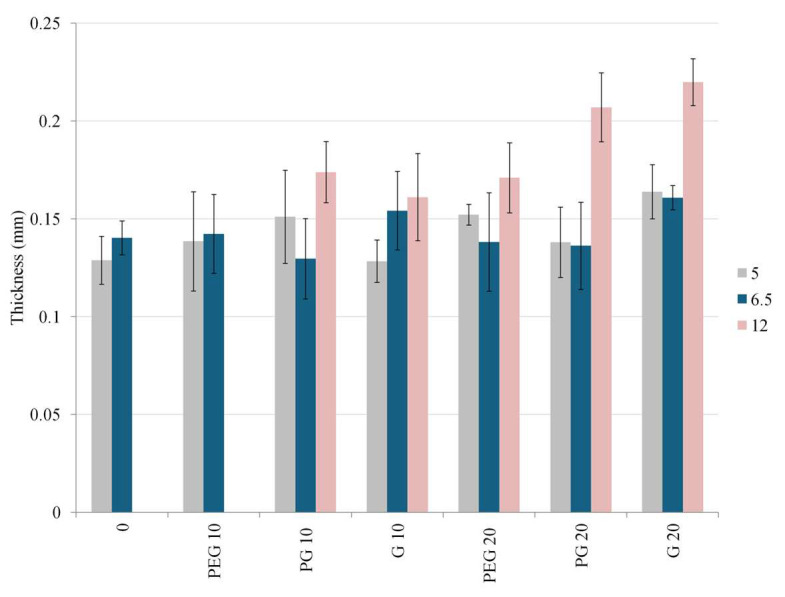
Thickness of films: 0—without added plasticiser; PEG, PG and G are films with added polyethylene glycol 400, propylene glycol and glycerol at 10% and 20% concentrations; 6.5, 5 and 12 are the pHs of the film-forming solutions before casting.

Film thicknesses shown in Figure 6 had mean values ranging from 0.128 mm to 0.220 mm. Although all films were produced by casting at a fixed ratio of mass to surface area, statistical analysis revealed certain differences in film thicknesses. Among the investigated parameters, the pH of the film-forming dispersion/solution had the most pronounced effect on the film thickness (F = 52.13, *p* < 0.001, η^2^ = 0.317), indicating that it is the factor that most strongly influences film structure formation. Films cast from the solution at pH 12 exhibit the highest thickness values, whereas the difference between the thicknesses of films cast from the cutin dispersions at pH 5 and 6.5 are not significant (*p* = 0.997).

This behavior may be associated with partial ester hydrolysis under strongly basic conditions, which can increase the free volume within the film and result in a less compact matrix. In addition, an increased number of negatively charged –COO^−^ groups, as detected by FTIR analysis, may promote electrostatic repulsion, potentially increasing the interchain distance, in agreement with the zeta potential results (Figure 1). Such structural changes could facilitate the incorporation of plasticisers, particularly glycerol, into the polymer network. Consequently, the increased hydrophilicity of the system may enhance water retention, contributing to the observed increase in film thickness [11,19].

The second most statistically significant factor affecting the film thickness is plasticiser concentration (*p* < 0.001), where a higher concentration of plasticiser leads to the formation of thicker films. This effect is most likely related to reduced intermolecular interactions, increased distance between chains, the flexibility of the matrix, and the free volume, as well as the moisture content, causing the matrix to expand.

Finally, although the influence of the type of plasticiser is the least pronounced, it remains significant (*p* = 0.037). A post hoc test indicates a significant difference in thickness only between films with glycerol and PEG. The estimated marginal means graph shows that glycerol produces thicker films than PEG, while films with PG do not differ significantly in thickness from the others. Glycerol has three –OH groups, which allows for extensive hydrogen bonding with the hydroxyl groups of fatty acids of cutin or the remaining polysaccharides and protein from the skin. For the same reason, this molecule is extremely hygroscopic and retains more water, further increasing the distance between the chains. Additionally, the glycerol molecule is small, unlike the PEG molecule, so its penetration through the film matrix is facilitated, leading to very good incorporation of glycerol into the film matrix [44].

Significant interactions were also observed between plasticiser type and concentration (*p* = 0.008), but the effect is not the same for all plasticisers and is most pronounced for glycerol. In addition, the significant interaction between pH and plasticiser type (*p* = 0.019) indicates that the influence of plasticisers depends on both their concentration and the pH conditions. At pH 5, these differences are relatively small, whereas at pH 12 they are more pronounced.

**Figure 7:**
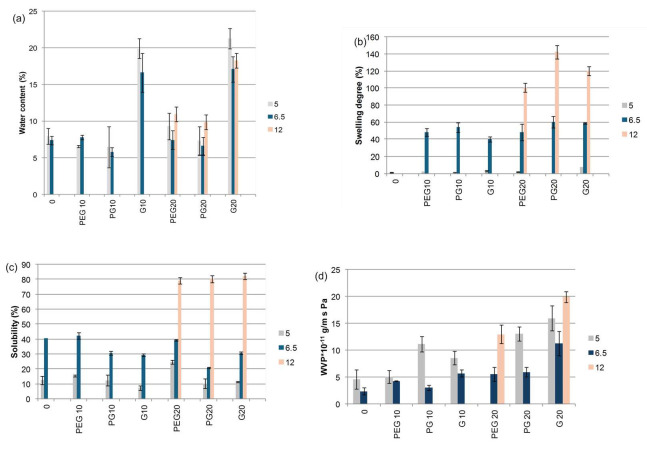
Properties of cutin isolate films: (**a**) water content (%), (**b**) swelling degree (%), (**c**) solubility (%), and (**d**) water vapor permeability.

Water content in films depends on the pH of the film-forming dispersion/solution (Figure 7a), but it is mainly influenced by the type of plasticizer (accounting for 85.3% of the variation in water content), and the effect of the plasticizer varies across different pH values. Post hoc analysis with interaction plots and estimated marginal means showed that the lowest water content occurs in films cast from the cutin dispersions at pH 6.5 (*p* < 0.05), while films cast at pH 5 and films cast from the solution at pH 12 have higher and mutually similar moisture content (*p* > 0.05). Regarding the most pronounced influence on moisture content, which is the type of plasticizer, films with glycerol have significantly higher water content than those with PEG and PG, while PEG and PG do not differ significantly from each other. The influence of concentration and the interaction between concentration and type of plasticizer on this property were not significant. This behavior can be attributed to the molecular structure of glycerol, a small molecule with three hydroxyl groups that provides high hydrophilicity and a strong ability to form hydrogen bonds with water. In contrast, propylene glycol contains two hydroxyl groups and one hydrophobic methyl group, while PEG 400 possesses a higher molecular weight and a linear polyether structure, resulting in lower water affinity than glycerol. Consequently, glycerol-plasticized films retain more water, which is consistent with their higher swelling and flexibility.

While the type of plasticizer was the main factor influencing water content, for film swelling (Figure 7b), the primary influence is attributed to the pH value of the film-forming dispersion/solution (accounting for up to 92.6% of the effect). As the pH increases, the degree of swelling also increases. Higher pH may lead to greater ionization of the functional groups, registered in the zeta potential values of the particles in film-forming dispersion/solution, as well as FTIR results. This increased ionization could make the polymer network more hydrophilic, thereby facilitating water penetration into the film matrix. In addition to this effect, the influence of the type of plasticizer and the interaction between these two factors is less pronounced but still significant. Glycerol-plasticized films exhibited the highest swelling degree, consistent with the higher hydrophilicity and water-binding capacity of glycerol compared with propylene glycol and PEG 400. Overall, the swelling behavior follows a trend similar to that observed for film thickness. The swelling values for the pH 6.5 cutin dispersion films are consistent with those obtained for starch and gelatin films coated with cutin [4], while the low degree of swelling observed in films cast at pH 5 is characteristic of a water-insoluble cutin material [11,28].

The results for solubility of the films in contact with water, the process that continues after swelling in contact with water, are entirely consistent with those for swelling (Figure 7c). This property is determined almost entirely by the pH of the film-forming dispersion/solution (η^2^ = 0.959). The type of plasticiser and its interaction with pH also have significant, though less pronounced, effects, while the concentration of the plasticiser has no significant influence. The use of PEG results in greater solubility compared to glycerol and propylene glycol, which do not differ significantly from each other. Films at pH 5 typically show low water solubility values for cutin-rich material. In contrast, a significant increase in solubility is observed at alkaline pH 12, where film solubility reaches values close to 80%. This behavior can be attributed to the deprotonation of residual carboxylic groups present in cutin oligomers, which increases the polarity and hydrophilicity of the material. This phenomenon is supported by FTIR analysis and is also reflected in the increase in the absolute value of the negative zeta potential on the surface of the particles in the film-forming dispersion/solution as the pH value of the suspension increases. Additionally, alkaline conditions may promote partial hydrolysis of ester bonds in the cutin structure, weakening the polymer network and facilitating film disintegration in water.

Regarding the water vapor permeability of the examined films, the dominant factor, accounting for more than half of the WVP variation (η^2^ = 0.532), is the pH of the film-forming dispersion/solution. Films cast from the dispersions at pH 6.5 exhibit the lowest water vapor permeability, followed by those from pH 5, with the highest values observed for films cast from the solution at pH 12. In addition to pH, the type of plasticiser also has a significant effect: films with glycerol show higher water vapor permeability than those with PEG, while other differences are not significant. The higher permeability of glycerol-containing films can be attributed to the small molecular size and high hydrophilicity of glycerol, which promotes water sorption and facilitates water vapor diffusion through the polymer matrix. The concentration of plasticiser is also a significant factor, as the higher amount of plasticiser, regardless of type, significantly increases WVP.

The WVP of the examined films is lowest in those cast at pH 6.5 and, except for the G20 films, falls within the range of (2.35–5.96) × 10^−11^ g/m s Pa (Figure 7d). Films cast at pH 5.0 with PEG10 also exhibit permeability within this range. These WVP values are up to 100 times lower than those reported for films based on pectin [45] and pullulan [45], and about 10 times lower than films based on starch–lentil flour, bean protein concentrate, chitosan, and lentil flour [46]. Compared to chitosan–cutin composite films, they are approximately 18 times lower [8], approximately half the value reported for methylcellulose films [46], while the values are similar to those for PLA [47] and cellophane films [46], but significantly higher than those for PET and PS, which are in the range of (0.11–0.67) × 10^−11^ g/m s Pa [47]. For comparison with the literature, only films evaluated under experimental conditions similar to those used in the present study were considered. Therefore, the reported values provide a reasonable basis for comparing the barrier performance of the different film systems.

**Figure 8:**
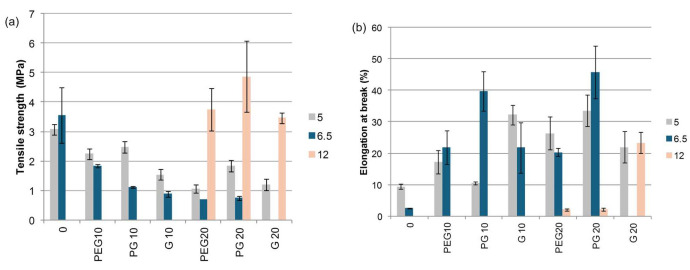
Mechanical properties of analyzed films: (**a**) tensile strength (MPa) and (**b**) elongation at break (%).

In both examined mechanical properties, tensile strength and elongation at break (Figure 8a,b), the most pronounced influence was exerted by the pH of the film-forming dispersion/solution. This effect was more pronounced for tensile strength. In addition to pH, the type of plasticiser also affected these properties, although to a lesser extent, and for elongation at break, a significant interaction between the type of plasticiser and pH was observed. Films cast from the solution at pH 12 exibited the highest tensile strength values, followed by those cast from the cutin dispersion at pH 5, whereas the lowest values were determined for films cast at pH 6.5. The maximum tensile strength values at pH 12 were accompanied by minimum values of elongation at break, while at pH 5 and pH 6.5, the elongation at break did not differ significantly.

Considering the combination of the two mechanical properties, it was observed that films pH 6.5/PEG10, pH 5/PEG10, pH 5/G10, pH 5/PG 20, and pH 12/G20 exhibit the highest values for both parameters. Other films proved either too brittle or too weak for practical use. The superior performance of these films suggests that an appropriate balance between polymer–polymer and polymer–plasticizer interactions was achieved. In particular, PEG 400 provided a favorable combination of strength and flexibility, while glycerol was effective at lower concentrations or in the more open pH 12 matrix, where its plasticizing effect enhanced flexibility without excessive loss of tensile strength. The mechanical properties of synthesized cutin films fall within the range reported for whey protein isolate films, as well as for a number of composite biopolymer films based on mixtures of fruit and vegetable residue flour, eggplant flour and corn starch, chickpea flour, Pinhão (*Araucaria angustifolia*) starch and flour, and plum oil cake films [46,48]. The mechanical properties of the investigated films are close to those of films obtained by blending sodium alginate and tomato pomace monomers in a 1:1 ratio [13] and are also very similar to those reported for synthetic polyaleurate film, a mimetic polymer of plant cutin [49]. Compared with PLA films, the tensile strength values are significantly lower, while the elongation at break values are comparable to some PLA films. In comparison with PET films, both properties of the investigated films are significantly lower [11,47].

**Figure 9:**
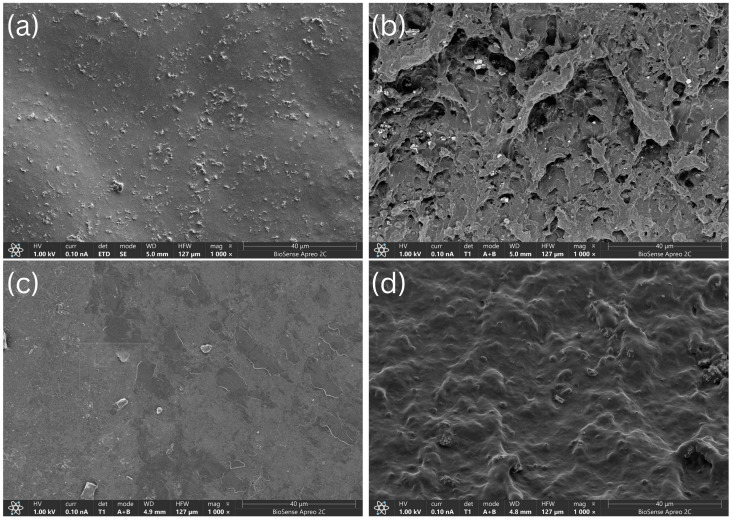
SEM images of cutin films cast from dispersion at pH 5: (**a**) pH 5, (**b**) cross-section of cutin film at pH 5, (**c**) pH 5/PEG20, (**d**) cross-section of cutin film pH 5/PEG20.

**Figure 10:**
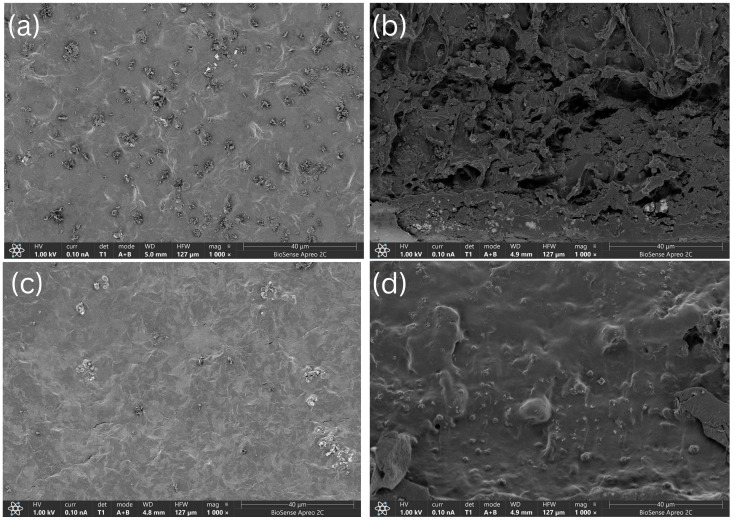
SEM images of cutin films cast from dispersion at pH 6.5: (**a**) pH 6.5, (**b**) cross-section of cutin film at pH 6.5, (**c**) pH 6.5/PEG20, (**d**) cross-section of cutin film pH 6.5/PEG20.

**Figure 11:**
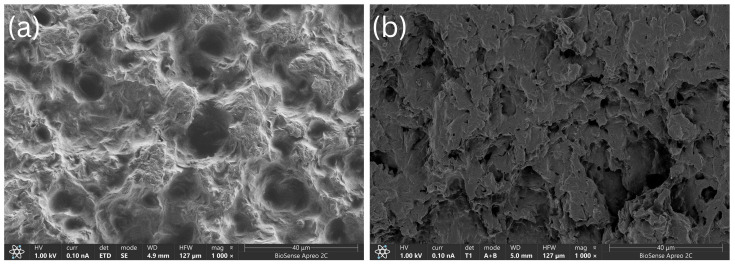
SEM images of cutin films cast from solution at pH 12: (**a**) film surface, (**b**) film cross-section.

For SEM analysis films obtained at different pH without plasticizer, as well as with 20% PEG, were chosen in order to analyze the influence of pH and plasticizer addition on the morphology of cutin films. The obtained results revealed that both parameters influenced films’ morphology on the film surface, as well as in the film internal, as shown in Figure 9, Figure 10 and Figure 11.

All tested films showed continuous matrix demonstrating good film-forming properties of isolated cutin under different casting procedures. However, significant differences in the degree of surface roughness and internal organization were observed.

The pH 5 film showed the smoothest and most homogeneous surface morphology, with only fine granular features and occasional micrometric protrusions. Although the surface appeared compact, cross-sectional images revealed a sponge-like interconnected network composed of irregular cavities separated by continuous polymer walls, probably originating from solvent evaporation.

The pH 6.5 films SEM images revealed more heterogeneous surfaces with increased roughness, frequent aggregates caught in the continuous matrix and shallow depressions. Although differences are apparent on the surface of films, when it comes to the appearance of the film’s cross-section, there appears to be no distinct difference between the pH 5 and pH 6.5 films.

The incorporation of 20% PEG modified the morphology. At pH 5 and pH 6.5, PEG produced a slightly rougher surface but promoted a more homogeneous internal organization, with fewer interconnected cavities and smoother fracture surfaces, suggesting enhanced chain mobility and structural rearrangement during drying. In this case, there is no spongy structure, but there is a compact, though granular, structure due to undissolved inclusions. No evidence of extensive phase separation or large PEG-rich domains was observed, indicating good compatibility between PEG and the cutin matrix.

SEM images of pH 12 film with 20% PEG20 revealed a highly porous morphology with numerous cavities, collapsed domains and thick matrix walls. The cross-section showed an open, irregular network rather than the relatively homogeneous cellular structure observed at lower pH values. This morphology is consistent with extensive ionization of carboxyl groups and partial hydrolysis/saponification of ester bonds, resulting in a highly hydrated and poorly packed polymer network.

The observed microstructural differences correlate well with the physicochemical and mechanical properties of the films. The progressively more open internal structure from pH 5 to pH 12 explains the increase in film thickness, swelling capacity and water solubility.

## 4. Conclusions

Proximate analysis and GC–MS analysis showed that the obtained isolate preserved the main structural characteristics of cutin and exhibited suitable potential as a film-forming material for the preparation of cutin-based films. The pH of the film-forming dispersion/solution represents the primary factor governing the properties of cutin dispersions and properties of the resulting cutin films. It directly influences the zeta potential of the cutin isolate, thereby affecting the particle size within the dispersion. A decrease in pH promotes more pronounced precipitation of the cutin isolate, leading to the formation of larger particles. The pH had a significant effect on all investigated film properties, most likely by influencing intermolecular interactions, partial breaking of ester bonds, and network density. The most pronounced effect was observed on the swelling, solubility, and tensile strength of the films, followed by water vapor permeability, elongation at break, and film thickness. The type of plasticiser used significantly influenced the following film properties: moisture content (the most pronounced influencing factor for this property), water vapor permeability (a pronounced influencing factor alongside pH), and thickness (statistically significant influence with all other factors). The concentration of the plasticiser had the greatest effect on water vapor permeability through the films, followed by film thickness. The interaction between the type and concentration of plasticiser had a significant effect only on film thickness, which was the only property significantly affected by each factor and each investigated factor interaction. The second investigated interaction, between pH and plasticiser, was significant for all properties except tensile strength, with the most pronounced effect on elongation at break. SEM analyses have shown that the microstructural organization of the films was strongly linked to their physicochemical and mechanical performance, with the progressively more open structure at higher pH values promoting increased film thickness, swelling capacity, and water solubility.

Considering the mechanical properties and water vapor permeability (WVP) of the films, the pH 6.5/PEG 10 and pH 5/PEG 10 films stand out as having good mechanical properties and low WVP compared to the others. Additionally, the pH 5/PEG 10 film shows lower sensitivity when in contact with water. If low WVP is not essential for the intended application, the pH 12/G20 film also exhibits good mechanical properties and, at the same time, high water solubility, which may be advantageous for certain applications.

## Figures and Tables

**Table 1:** Summary of the prepared films, including explanations for samples excluded from further analysis owing to insufficient mechanical properties.

Plasticizer Type	Plasticizer Concentration (%)	pH 5	pH 6.5	pH 12
/	0	Analyzed	Analyzed	Not analyzed—excessively brittle
G	10	Analyzed	Analyzed	Not analyzed—excessively brittle
	20	Analyzed	Mechanical properties not analyzed due to film stickiness	Analyzed
PG	10	Analyzed	Analyzed	Not analyzed—excessively brittle
	20	Analyzed	Analyzed	Analyzed
PEG	10	Analyzed	Analyzed	Not analyzed—excessively brittle
	20	Analyzed	Analyzed	Analyzed

**Table 2:** Results of ANOVA for the effects of pH, plasticizer type and concentration on the physicochemical and mechanical properties of the films.

Property	Factor	F	*p*	η^2^
Thickness	pH	52.13	<0.001	0.317
	plasticizer	3.36	0.037	0.020
	pH×plasticizer	3.01	0.019	0.037
	plasticizer	5.29	0.006	0.041
	concentration	25.66	<0.001	0.101
	plasticizer×concentration	4.89	0.008	0.038
Water content	pH	7.45	0.004	0.045
	plasticizer	140.26	<0.001	0.853
	pH×plasticizer	3.09	0.038	0.038
	plasticizer	99.874	<0.001	0.874
	concentration	4.015	0.057	0.018
	plasticizer×concentration	0.351	0.708	0.003
Swelling degree	pH	292.26	<0.001	0.926
	plasticizer	4.55	0.021	0.014
	pH×plasticizer	3.12	0.033	0.020
	plasticizer	0.745	0.484	0.049
	concentration	0.290	0.595	0.010
	plasticizer×concentration	0.208	0.814	0.014
Solubility	pH	739.94	<0.001	0.959
	plasticizer	10.31	<0.001	0.013
	pH×plasticizer	5.34	0.004	0.014
	plasticizer	0.3394	0.716	0.024
	concentration	3.9961	0.057	0.139
	plasticizer×concentration	0.0413	0.960	0.003
WVP	pH	21.0	<0.001	0.532
	plasticizer	140.26	<0.001	0.853
	pH×plasticizer	3.09	0.038	0.038
	plasticizer	4.82	0.014	0.150
	concentration	15.48	<0.001	0.240
	plasticizer×concentration	2.64	0.086	0.082
TS	pH	125.11	<0.001	0.800
	plasticizer	4.94	0.012	0.032
	pH×plasticizer	2.41	0.064	0.031
	plasticizer	0.297	0.745	0.012
	concentration	1.369	0.248	0.028
	plasticizer×concentration	0.535	0.589	0.022
EB	pH	35.82	<0.001	0.471
	plasticizer	3.31	0.046	0.043
	pH×plasticizer	7.74	<0.001	0.203
	plasticizer	1.490	0.236	0.057
	concentration	0.347	0.558	0.007
	plasticizer×concentration	1.662	0.201	0.063

**Table 3:** Proximate composition of tomato cutin isolate.

Component	Content *
Moisture (% wb)	2.54 ± 0.01
Ash (% dw)	0.18 ± 0.05
Fat (% dw)	12.12 ± 0.42
Proteins (% dw)	7.53 ± 0.55
Fibers (% dw)	17.15 ± 1.12
Residual fraction (% dw)	63.02 ± 1.32

* mean ± standard deviation.

**Table 4:** GC–MS profile of compounds released by methanolysis of tomato cutin isolate.

Compound	RT (min)	Identification	% (mean ± SD)
		Fatty acids/fatty acid derivatives	
1	9.064	16:0 ME	0.89 ± 0.18
2	10.264	16:0 TMSE	0.78 ± 0.12
3	10.786	18:2 ME	1.54 ± 0.25
4	10.853	18:1 ME	0.85 ± 0.15
5	11.119	18:0 ME	0.27 ± 0.07
6	12.252	18:1 TMSE	0.73 ± 0.13
7	12.586	18:0 TMSE	0.83 ± 0.14
8	18.529	22:1 TMSE	0.29 ± 0.37
9	22.718	24:0 TMSE	0.42 ± 0.29
		Mono-hydroxy fatty acids	
10	13.13	16-Hydroxy hexadecanoic acid	2.60 ± 0.35
		Di- and tri-hydroxy fatty acids (cutin-related monomers)	
11	16.174	10,16-Dihydroxy hexadecanoic acid	70.02 ± 4.25
12	21.618	9,10,18-Trihydroxy octadecanoic acid	1.13 ± 0.01
		Other identified compounds	
13	6.82	*p*-Coumaric acid	0.68 ± 0.10
14	7.797	*p*-Coumaric acid	0.95 ± 0.14
15	12.708	1,16-Hexadecanedioic acid	0.90 ± 0.14
16	18.307	1-Monopalmitoylglycerol (TMS ether)	3.51 ± 0.09
17	21.951	1-Monostearin (diTMS)	3.67 ± 0.03
		Non-identified peaks (NI)	
		NI	9.94 ± 2.69
TOTAL			100.00

ME: methyl ester; TMSE: trimethylsilyl ester/derivative; NI: non-identified. Values are expressed as mass percentage (%), mean ± SD (n = 4).

**Table 5:** Influence of cutin isolate concentration on the mean volume weighted particle diameter, (d_4,3_), at pH 5 and pH 6.5.

c/%	d_4,3_ (pH 5)/µm	d_4,3_ (pH 6.5)/µm
1	10.95	13.83
5	49.82	40.14
10	96.2	77.4

## Data Availability

The original contributions presented in this study are included in the article. Further inquiries can be directed to the corresponding author.
